# Application of autologous platelet-rich gel formed by calcium gluconate combined with hormone therapy for endometrial repair after hysteroscopic transcervical resection of adhesion surgery and successful pregnancy: case report and literature review

**DOI:** 10.3389/fmed.2024.1436089

**Published:** 2024-09-18

**Authors:** Yunying Li, Yingxue Han, Xiaojuan Su, Junjuan Cao, Junxia Liu, Wenjuan Zhang

**Affiliations:** ^1^Department of Gynecology and Obstetrics, Shijiazhuang People's Hospital, Shijiazhuang, China; ^2^Transfusion Department, Shijiazhuang People's Hospital, Shijiazhuang, China

**Keywords:** intrauterine adhesion, autologous platelet-rich gel, calcium gluconate, endometrial repair, pregnancy, case report

## Abstract

**Introduction:**

Intrauterine adhesion (IUA), a common gynecological disease, is mainly caused by traumatic or infectious factors that lead to basal endometrial layer physiological repair disorders. IUA is mostly treated via hysteroscopic transcervical resection of adhesion and although it can restore uterine cavity shape, its endometrial repair effectiveness is limited. The figures showed that after surgery, patients with IUA have a high recurrence rate. Therefore, quick endometrial damage repair is key to successful treatment.

**Case presentation:**

A 34-year-old patient visited our hospital after experiencing amenorrhea for 4 months following an induced abortion and had a fertility requirement. Based on the American Fertility Society intrauterine scores, the patient was diagnosed with moderate IUA. She underwent transcervical resection of adhesion, followed by autologous platelet-rich gel intrauterine perfusion and periodic estrogen–progesterone treatment for three menstrual cycles. No complications developed during treatment and the patient’s endometrium was significantly repaired, with successful pregnancy being achieved.

**Conclusion:**

Autologous platelet-rich gel promoted endometrial repair and acted as a mechanical barrier to prevent intrauterine adhesion. This approach May offer new insights into IUA treatment.

## 1 Introduction

Intrauterine adhesion (IUA), which is also known as Asherman’s syndrome, is a common gynecological disease. Its major causes include multiple intrauterine operations, such as induced abortion and dilatation and curettage, as well as infection, which can damage the endometrium’s basal layer. Failure of normal endometrial regeneration and damage repair leads to fibrosis and adhesion formation, eventually leading to a partial or complete closure of the uterine cavity (1). The main IUA symptoms include reduced menstrual volume and even amenorrhea, secondary infertility, and miscarriage (2). Severe IUA recurrence rates after hysteroscopic transcervical resection of adhesion (TCRA), the preferred IUA treatment method, are as high as 62.5% (3). This is because although surgery can improve uterine cavity morphosis, it May aggravate endometrial damage. Currently, postoperative IUA recurrence is often prevented using drug therapy, mechanical barriers, or anti-adhesion agents. However, these strategies are associated with long treatment cycles, limited efficacy for moderate-to-severe IUA, and the risk of complications (1). Platelet-rich plasma (PRP) is a platelet concentrate extracted from fresh autologous blood, and after its activation, platelet *α*-granules release various growth factors, including vascular endothelial growth factor and platelet-derived growth factor, and cytokines, which promote cell proliferation, angiogenesis, and matrix remodeling (4). Moreover, PRP activation converts soluble fibrinogen into insoluble fibrin, thereby forming an autologous platelet-rich gel (APG), which prolongs the retention and action time of active substances (5). APG May prevent intrauterine adhesion by a offering mechanical barrier function. Calcium gluconate-activated PRP has been shown to accelerate diabetic wound healing and to possess neuroprotective and neuroregenerative effects (6, 7). This report describes for the first time that the postoperative application of calcium gluconate-activated APG in patients with IUA. This patient’s menses has recovered and then she is pregnant.

## 2 Case presentation

**Figure 1:**
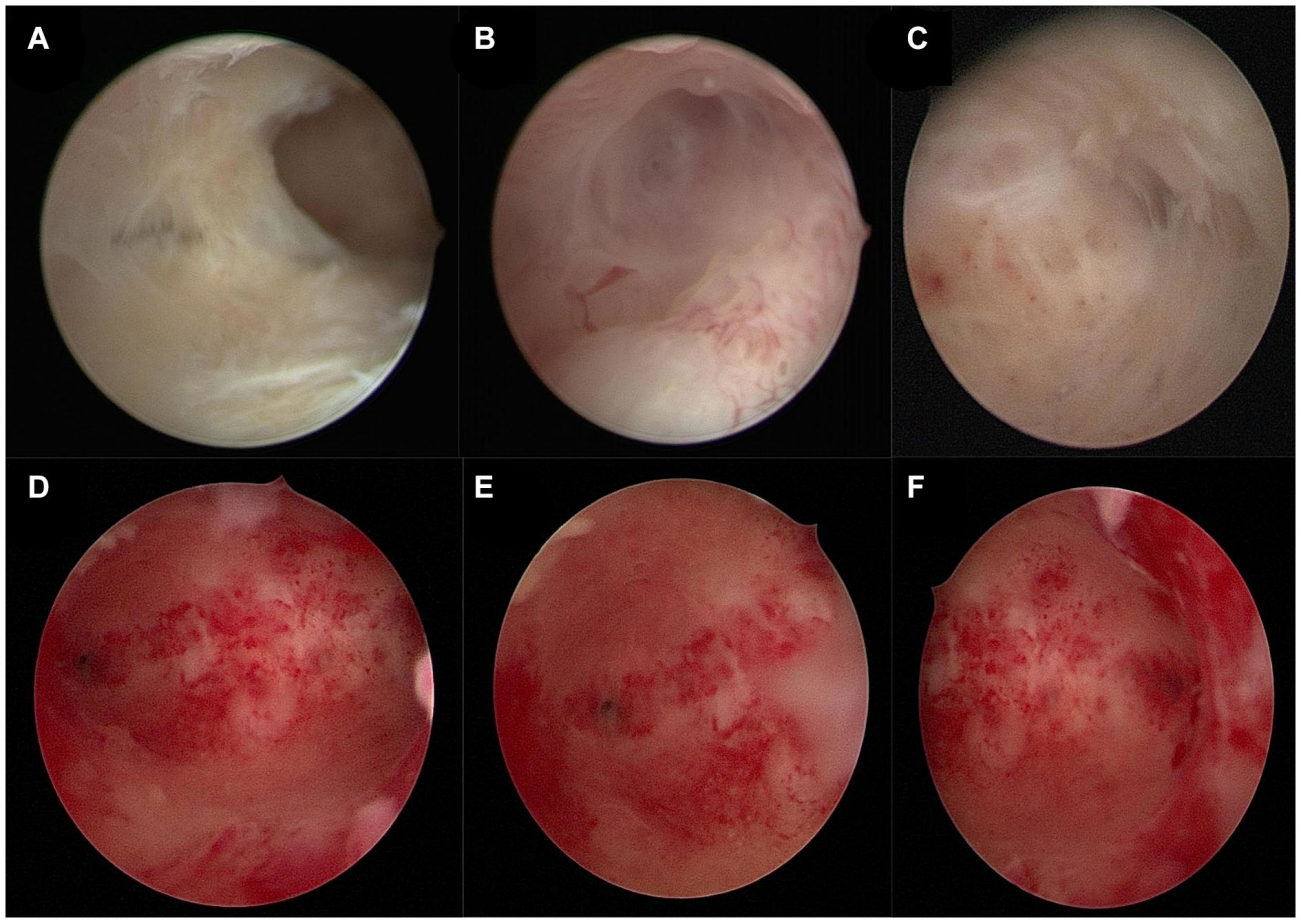
**(A–C)** Pre-surgery hysteroscopic patient examination images. **(A)** Adhesion of the uterine cavity’s right wall. **(B)** Right fallopian tube opening. **(C)** Left fallopian tube opening. **(D–F)** Post-treatment hysteroscopic patient examination images. **(D)** Normal uterine cavity. **(E)** Right fallopian tube opening. **(F)** Left fallopian tube opening.

In February 2023, a 34-year-old patient visited our hospital after experiencing amenorrhea for four months following an induced abortion and had a fertility requirement. The patient had previously undergone two natural births and three induced abortions. Transvaginal ultrasound revealed that the endometrium had grown unevenly, at a thickness of 0.1–0.29 cm (average: 0.20 cm). However, a hormone test did not reveal significant abnormalities. Hysteroscopy examination indicated intrauterine adhesions immediately (Figures 1A–C) and based on American Fertility Society intrauterine scores, the patient was diagnosed with moderate IUA (8).

## 3 Surgical procedure

**Figure 2:**
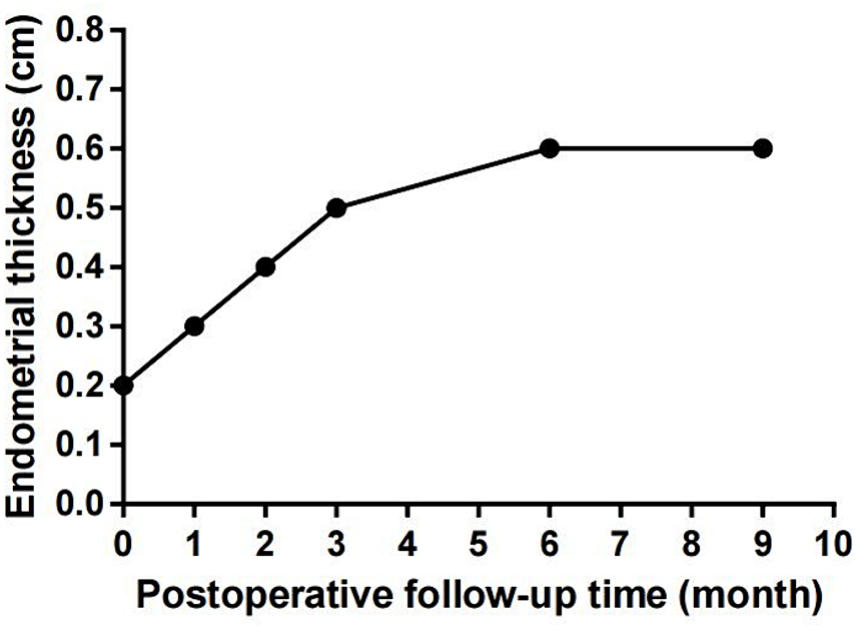
The patient’s endometrial thickness increased gradually during the treatment, and there was no significant change during post-treatment follow-up.

PRP was extracted using a blood cell separator (NGLXCF3000, Nigale Biomedical, Sichuan, China), followed by hysteroscopic TCRA the next day. To prepare APG, 10% calcium gluconate was added to the PRP at a 1:9 ratio, mixed, and immediately administered into the uterine cavity via perfusion (9), and the patient was asked to remain in the perfusion position for 15–20 min. After the surgery, the patient took 4 mg of estradiol valerate daily and underwent APG intrauterine perfusion every 7–10 days. The patient started menstruating in April 2023 at about 1/3 to 1/2 of her normal menstrual volume. Hysteroscopy examination was performed on the first day after menstruation. The images revealed improved uterine cavity morphology without adhesion and that the endometrium was uniform (Figures 1D–F). Intrauterine APG perfusion was done on the first and eighth days after menstruation, with periodic treatment for three menstrual cycles using estrogen (4 mg of estradiol valerate daily for 21 days) and progesterone (20 mg of dydrogesterone daily for the last 10 days). During the mid-luteal phase, endometrial thickness increased gradually to 0.6 cm (Figure 2). No complications were observed during the treatment and the patient got pregnant in November 2023.

## 4 Discussion

IUA is one of the common gynecological diseases. The main reason for its formation is the repair dysfunction of endometrial cells after injury. IUA patients often seek medical advice due to hypomenorrhoea, amenorrhea and infertility. Hysteroscopic TCRA, as the preferred surgery for IUA patients, can improve the morphosis of uterine cavity. But the recurrence rate of postoperative adhesion is high. Therefore, adopting effective methods to repair the endometrium and prevent recurrence after surgery is also crucial in the treatment of IUA. In recent years, the role of PRP cell therapy in regenerative medicine has been gradually explored. Autologous PRP is easily obtainable and it does not carry disease transmission or immune rejection risks. Through growth factor and cytokine release, PRP promotes cell proliferation and anti-inflammation (10, 11). In the clinical application of obstetrics and gynecology, PRP can relieve vulvovaginal atrophy symptoms and urinary incontinence stress in genitourinary syndrome during menopause, as well as prevent uterine niche formation after cesarean section (12–14). PRP improves endometrial thickness and receptivity by increasing angiogenesis-related markers and modulating immune cells and the microbiome. Consequently, PRP improves menstruation and pregnancy outcomes in patients with thin endometria and endometritis (15–17). A study on an IUA mouse model revealed that intrauterine PRP injection improves endometrial stromal cell migration, embryo implantation, and live birth rates. Currently, there are few clinical data on the application of PRP in postoperative patients with IUA. Javaheri et al. (18) demonstrated that there was no significant difference in menstrual improvement and prevention of adhesion recurrence between IUA patients who received 1 mL PRP single intrauterine injection after surgery and those who did not receive it. Aghajanova et al. (19) also confirmed that estrogen therapy combined with 0.5-1 mL PRP single intrauterine injection after IUA surgery showed no changes in endometrial thickness and pregnancy rate of patients compared to the estrogen only treatment group. The emergence of these results May be related to the liquid properties of PRP, which cannot stay in the uterine cavity for a long time to fully exert its effect.

It is reported that after PRP activation, platelets are degranulated, thereby releasing several growth factors (5). Fibrinogen activation can form a gel that provides a temporary cell proliferation and migration scaffold. At the same time, bioactive factors can be protected from proteolytic enzyme degradation and be released slowly (20, 21). Common PRP agonists include calcium gluconate, calcium chloride, and thrombin. Considering its heterogeneity, thrombin use as a PRP activator May pose certain clinical application risks (22). Growth factor and cytokine release in 10% calcium gluconate-activated PRP gels is similar to thrombin’s (23, 24) and is associated with a higher cell proliferation rate when compared with inactive PRP (25). When compared with calcium chloride, calcium deposition is rare in gels formed by calcium gluconate-activated PRP (23). Calcium gluconate-activated PRP gels have been used as diabetes skin ulcer dressing to promote wound healing (6). Moreover, it is reported that calcium gluconate-activated PRP gels can promote spiral ganglion neuron neuroregeneration and neuroprotection *in vitro* (7). Injecting calcium gluconate-activated PRP gels into the ovaries of patients with premature ovarian insufficiency is reported to improve ovarian function (26). Additionally, the gel formed by calcium salt retracted more slowly, which is beneficial for barrier properties and adhesion prevention (23).

In this report, we describe the therapeutic effects of a calcium gluconate-activated autologous platelet-rich gel in combination with hormone therapy in patients with IUA after adhesiolysis. First, to ensure cellular component stability, we used a blood cell separator to extract PRP. Second, because calcium gluconate-activated PRP gels at 37°C after about 10 min (9), the patient was required to maintain the perfusion position for 15–20 min. Furthermore, for unresistant perfusion without cervical dilatation, a tube was used for hydrotubation to reduce patient discomfort. The time interval for APG perfusion was determined based on the platelet metabolic cycle. There were no complications during the treatment process. Moreover, the patient’s endometrial thickness increased steadily, and she became pregnant. Because endometrial damage-associated risks, such as miscarriage and premature birth remain, close follow-up is required. Because there is no consensus about the protocol for APG use for endometrial repair, this case report May provide some ideas. However, randomized controlled trials are needed to validate the effectiveness and safety of this case’s protocol, as well as to evaluate long-term APG effects. Moreover, simultaneous cell and animal experiments are needed to determine the underlying mechanisms.

## 5 Conclusion

This case indicates that in patients with moderate IUA, autologous platelet-rich gels have the potential to prevent postoperative recurrence, repair the endometrium, and increase the probability of pregnancy. It is important to provide timely and effective treatment measures for patients with intrauterine adhesions after surgery for preventing recurrence. However, larger clinical datasets are needed to confirm this method’s safety and effectiveness. Nonetheless, this approach May offer new IUA treatment ideas.

## Data Availability

The original contributions presented in the study are included in the article/supplementary material, further inquiries can be directed to the corresponding author.
